# Preservation of intact adult rat photoreceptors in vitro: Study of dissociation techniques and the effect of light

**Published:** 2009-01-09

**Authors:** Astrid Zayas-Santiago, Jennifer J. Kang Derwent

**Affiliations:** Department of Biomedical Engineering, Pritzker Institute of Biomedical Science and Engineering, Illinois Institute of Technology, Chicago, IL

## Abstract

**Purpose:**

Intact adult photoreceptors in culture can be a valuable tool in the search of therapies for retinal degenerations. The major challenge in this technique is that photoreceptors undergo an alteration in cytoarchitecture and loss of outer segment during the cell culture process. This study compared techniques for the isolation of photoreceptor cells from adult rat retinas to determine which technique yields the highest percent of structurally well preserved cells in vitro. In addition, the role of light exposure during the dissociation and culture process was investigated to minimize photoreceptor cell deformation over time in culture.

**Methods:**

Photoreceptor cells from adult rat retinas were isolated and quantified using three dissociation techniques: enzymatic dissociation with gentle pipeting; enzymatic dissociation with gentle pipeting and centrifugation; and non-enzymatic dissociation with gentle pipeting. To evaluate the effect of light exposure on cell deformation, we performed dissociations and cell seeding both in dark- and light-adapted conditions and measured the deformation of photoreceptors over a 12 h period right after dissociation. Cell viability in both conditions was evaluated after 4 and 7 days in culture. Preservation of cell structure in culture was assessed by immunofluorescence labeling of cells with anti-rhodopsin and 4',6-diamidino-2-phenylindole (DAPI) nuclear staining.

**Results:**

An enzymatic technique followed by gentle pipeting or mechanical trituration yielded the highest number of intact elongated photoreceptors right after dissociation. Data suggested that centrifugation after the dissociation contributed to cell deformation immediately after isolation. Immunohistochemistry results showed that cells had deformed into a circular shape by 2 days after seeding. However, photoreceptors isolated in dark conditions maintained their elongated shape, even 7 days after seeding. Performing experiments in dark also promoted a higher number of cells to remain viable with time.

**Conclusions:**

The current study demonstrated the importance of proper isolation techniques to obtain the maximum amount of intact photoreceptor cells. The data suggested that a gentle dissociation technique, consisting of enzymatic treatment followed by moderate pipeting of the retinal tissue, may be the key to obtain a high number of intact or structurally preserved photoreceptors. Furthermore, isolation and cell culture procedures performed under dark conditions may facilitate to maintain high number of elongated photoreceptor cells in vitro.

## Introduction

Utilization of isolated photoreceptor cells can be a valuable tool in the field of vision science and ophthalmology. It offers a simplified environment for studies that require individual cell assessment or controlled intercellular communication. Areas such as pharmacology and mechanisms of growth factors [1-4], receptor and signaling pathways [5,6], gene expression [7], and neural regeneration [8-11] have significantly benefited from utilization of isolated photoreceptor cells. Isolated photoreceptors are also used in transplantation techniques aiming to rescue and replace degenerated photoreceptors [12-14]. These applications are critical for the understanding of photoreceptor cell function, inherited retinal diseases, and the search of prospective therapies. Most of them require isolation of individual photoreceptor cells in high numbers, and many require cell culturing techniques that facilitate studies over long time periods.

Adult photoreceptor cells have been isolated and cultured since the late 1970s [15,16]. Due to the early belief that mature cells lack the ability to survive in artificial conditions, most in vitro models relied on embryonic or early postnatal cells [4,17]. However, MacLeish and colleagues demonstrated that adult photoreceptors when cultured on permissive substrates can be successfully maintained for several weeks [18]. In addition, adult photoreceptors in culture have been shown to regenerate neurites and make connection with neighboring cells [8,9,11], continue expressing typical synaptic vesicle proteins [19], and continue to be functional [20-22]. These findings have prompted us to reconsider fully differentiated adult photoreceptors maintained in vitro as model for retinal studies and the possibility of employing these cells in transplantation techniques [17].

In spite of these promising findings, there are still major challenges for successfully culturing adult photoreceptors. The major limitation is the damage in cell structure suffered during the cell culture process. Photoreceptors have a delicate structure in which the outer segment is connected to the inner segment by a thin cilium and, similarly, inner segment to the cell body by a thin outer fiber [23]. Right after dissociation, the cell density is mostly populated with rounded photoreceptors that lack the synaptic terminal, outer segment, or both [9]. It appears that the dissociation methods commonly used may be destructive and responsible for the loss of outer segment and synaptic pedicles. Furthermore, photoreceptors that remain intact after dissociation tend to slowly deform with time. Long-term studies have shown that the photoreceptor alters its elongated outer segment into circular shape in cell culture [10,24,25]. In these studies, although rounded photoreceptors do express rhodopsin, they did not possess a well developed outer segment. Townes-Anderson et al. [22] described this occurrence as a slow fusion of the base of the outer segment with the cytoplasm of the inner segment. Correspondingly, outer segments are also observed to be small, distorted, and even absent in cells transplanted as suspension into retinal degeneration models [12,13,26-29].

To date, there is no consensus on what process governs the morphological changes suffered by isolated intact photoreceptors. It was suggested that perhaps it is due to the absence of the retinal pigment epithelium [15,30], a key factor needed for the formation and maintenance of the outer segment. The slow and poor attachment of adult photoreceptors to standard culture surfaces [23] and the lack of interactions with glial cells [10] have been implied. To obtain reliable results from studies performed with isolated photoreceptors, the cells should maintain their elongated in vivo shape and structure. The main goals of this study were to perform a quantitative study of dissociation techniques to obtain intact adult photoreceptor cells for seeding in culture and investigate methods to minimize the outer segment loss as well as deformation of the cells over time in culture. We first revisited early studies by Sarthy and Lam [16], neuronal dissociation techniques developed by Brewer [31], and existing retinal dissociation techniques discussed by Romano and Hicks [17] to perform a quantitative analysis of outer segment loss and deformation. Then, we examined the effect of light exposure on outer segment deformation during the isolation and culture process. Photoreceptors respond to light by closing ionic channels in the outer segment which consequently causes the cell to hyperpolarize, creating a potential gradient across its membrane. We hypothesized that this potential gradient may be involved in the membrane deformation observed in cells isolated in standard light conditions.

## Methods

### Photoreceptor dissociation techniques

All animal procedures adhered to the Association for Research in Vision and Ophthalmology (ARVO) statement for the Use of Animals in Ophthalmic and Vision Research and according to institutional animal care and use guidelines. The animals were housed in a standard cage in a 12 h light-dark cycle room and had access to food (standard rat chow) and water freely. The eyes were obtained from Long-Evans pigmented rats (22–25 days old; Charles River Laboratories, Wilmington, MA) that were sacrificed by CO_2_ asphyxiation. Eyes were enucleated with minimum extraneous tissue, rapidly immersed in 70% ethanol, suspended in cold Hybernate A medium (HybA; Brain Bits LLC, Springfield, IL) and transferred to the laboratory for immediate isolation of the retina. Each retina was detached from the eyecup while suspended in cold HybA. It was then transferred to a tube with 1ml HybA, and warmed up in a water bath at 37 °C for 8 min. The dissociation of photoreceptors from the retinas was studied using three different techniques: two enzymatic (papain treatment) and one non-enzymatic (mechanical trituration only).

#### Enzymatic dissociation with gentle pipeting [16] (Protocol A)

The retinas were warmed to 37 °C. HybA was aspirated and 1 ml papain (0.06 mg/ml [33.4 U/mg]; Worthington Biochemical, Lakewood, NJ) prepared in HybA was added to each retina tissue for 20 min at 37 °C. Papain was not activated with L-cysteine, which according to Brewer [31], can be cytotoxic; it was only pre-warmed at 37 °C for 30 min. Tissues were shaken gently twice every 10 min. Papain solution was aspirated and 2 ml of 2% fetal bovine serum (FBS; Mediatech, Manassas, VA) in HybA was added for 5 min to stop the enzymatic reaction. FBS solution was aspirated and HybA supplemented with 1:50 B27 (Invitrogen, Carlsbad, CA) and 0.5 mM L-glutamine (HybA+; Sigma-Aldrich, St. Louis, MO) was added to the tissues. To dissociate the cells, the papain-treated retinas were manually pipetted (5 to 6 times) using wide-bore 1,000 µl tips (USA Scientific, Ocala, FL) while suspended in HybA+ medium. The tissues were allowed to settle for 2 min and the supernatant containing the dissociated cells was collected.

#### Enzymatic dissociation with gentle pipeting and centrifugation [4,32] (Protocol B)

This technique followed the same initial steps used in Protocol A, however, adding a final centrifugation step. Once the final suspension of dissociated cells was obtained, the cell suspension was then centrifuged at 129x g for 5 min, the supernatant was discarded, and the cell pellet containing the dissociated cells was resuspended in the HybA+ medium and collected.

#### Non-enzymatic dissociation with gentle pipeting [33] (Protocol C)

Once the retinas were warmed to 37 °C, HybA was aspirated and the tissues were resuspended in HybA+. To dissociate the cells, the retinas were manually pipetted (5 to 6 times) using wide-bore 1,000 µl tips while suspended in HybA+ medium. The tissues were allowed to settle for 2 min and the supernatant containing the dissociated cells was collected.

### Intact photoreceptor cell counting

**Figure 1:**
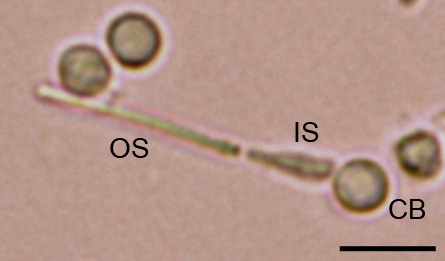
Intact photoreceptor dissociated from an adult rat retina. The image shows the morphologic features of rod photoreceptors: outer segment (OS), inner segment (IS), and cell body (CB). The synaptic pedicle is missing. The scale bar represents 10 μm.

The dissociated cells obtained after each dissociation technique were seeded at a density of 2×10^5^ cells/cm^2^ in 24 well culture plates. The amount of intact photoreceptor obtained with each technique was determined in triplicate (three wells per dissociation technique). A cell was identified as an intact photoreceptor based on typical morphological features of outer segment, inner segment, and cell body (Figure 1). Transmitted light images were obtained under a 40× objective (Olympus 1X71 inverted microscope; Olympus, Tokyo, Japan) in 30 adjacent fields. Results are expressed as the percent (%) of intact photoreceptors to total nuclei observed per image. Images were acquired with a digital color camera (Olympus DP70; Olympus, Tokyo, Japan) and analyzed using Image J software (NIH Image J 1.41; National Institutes of Health, Bethesda, MD).

### Photoreceptor cell culture

For photoreceptor cell culture, the HybA+ medium used for cell dissociation and final seeding was substituted with Neurobasal Medium A (NBA; Invitrogen) supplemented with 1:50 B27 (Invitrogen) and 0.5 mM L-glutamine (Sigma Aldrich; NBA+). The cells obtained after the dissociation techniques were seeded onto glass coverslips (round 8 mm diameter No. 1.5 German glass; Electron Microscopy Sciences, Hatfield, PA) placed on 24 well culture plates at a density of 2×10^5^ cells/cm^2^ and incubated at 37 °C under a humidified atmosphere of 95% air and 5% CO_2_. Cells were allowed to attach overnight before the first change in medium. Then, medium was changed every 3 to 4 days.

Coverslips used as substrates were pre-coated with wheat germ agglutinin (WGA) lectin to promote optimal attachment [25]. They were incubated for 2 h at 37 °C with 2.5 μg/cm^2^ anti-WGA directed against WGA lectin (Vector Laboratories, Burlingame, CA) diluted in 25 mM bicarbonate buffer, pH 8, that contained 0.9% NaCl and 2 mg/ml BSA (BSA, Fraction V; Sigma-Aldrich). After three washes with warm bicarbonate buffer, they were consequently coated with 5 μg/cm^2^ WGA lectin (Vector Laboratories) diluted in bicarbonate buffer. After 2 h at 37 °C, coverslips were washed with warm phosphate-buffered saline (PBS; 0.144 g/l KH_2_PO_4_, 9.00 g/l NaCl, 0.795 g/l Na_2_HPO_4_ [anhydrous]; pH 7.4) and stored in 0.2% BSA in PBS until used.

#### Effect of light on dissociation technique

Photoreceptor cells were dissociated using Protocol A either under dim-red light (dark-adapted) or under room light (light-adapted). They were seeded at a density of 2×10^5^ cells/cm^2^ onto 35 mm suspension dishes. The elongation and shape of intact photoreceptors after dissociation was analyzed by measuring their circularity ratio, using Image J, with the following equation:

$${\text{CR=4π(Area/Perimeter}}^{\text{2}}\text{)}$$

A value of 1 indicated a perfect circular cell, while values closer to 0 indicated an elongated cell. For each condition, experiments were performed in triplicate and with more than 30 intact photoreceptors analyzed per time point for up to 12 h. For dark-adapted studies, rats were kept in the dark for 10 h before the experiment. Enucleation, dissociation, and imaging were performed under a dim-red light. The microscope lamp was filtered with a deep red wratten filter (Kodak #29; Edmund Optics, Barrington, NJ). In light-adapted studies, enucleation, dissociation, and imaging were performed under room light conditions and using a standard microscope lamp. The temperature of the cell medium was maintained at 37 °C using a microscope stage warmer.

### Viability of photoreceptors with time

Photoreceptor cells were dissociated using Protocol A under dark-adapted or light-adapted conditions and seeded at a density of 1.5×10^6^ cells/cm^2^ (4.5×10^5^ cells/well) in a 96 well tissue culture plate (100 μl/well) while suspended in NBA+ medium. To test the viability of cells, 10 μl of CellQuanti-Blue^TM^ reagent (CellQuanti-Blue^TM^ cell viability assay kit; BioAssay Systems, Hayward, CA) was added to the media in each well, and the plate was incubated at 37 °C overnight. The assay consists in the metabolic reduction of the non-fluorescent blue dye (resazurin) by living cells into a highly fluorescent product (resorufin). Approximately 16 h after adding the reagent, the fluorescence intensity was measured using a plate reader (Wallac Victor^3^V; 530 nm excitation filter, 590 nm emission filter; PerkinElmer, Waltham, MA). Since only viable cells can reduce the reagent, the fluorescence intensity observed represents measure of the amount of viable cells [34]. The amount of viable cells was measured at times: 0 (total photoreceptors initially seeded), and 4 and 7 days after initial seeding. Each experiment was performed three times, with four replicates per experiment.

### Immunohistochemistry

Cultured cells were fixed in 4% paraformaldehyde prepared in PBS for 15 min, rinsed with PBS, and then permeabilized with 0.1% Triton X-100 for 15 min. After additional rinsing with PBS, the cells were incubated for 30 min with Image-iT™ FX signal enhancer (Invitrogen) to reduce background staining. Nonspecific labeling was blocked by rinsing cells again and incubating for 30 min in 1% BSA in PBS (Buffer B). The cells were then stained by incubating with 1.5 μg/ml mouse monoclonal antibody anti-rhodopsin (RET-P1; Millipore, Billerica, MA) in Buffer B for 2 h at room temperature. After rinsing with Buffer B, the cells were incubated with a fluorescent secondary antibody, goat anti-mouse IgG, Alexa Fluor 488 (5 μg/ml; Invitrogen) and with 5 μg/ml of the nuclear marker 4’,6’-diamino-2-phenylindole (DAPI; Sigma Aldrich) in Buffer B for 45 min at room temperature. Finally, the cells were washed with PBS, mounted, and imaged by epifluorescence microscopy (Olympus BX61; Olympus, Tokyo, Japan).

### Statistical analysis

All results are expressed as the standard error of the mean (SEM). Statistical comparisons were performed using Student’s *t*-test. P values <0.05 were considered statistically significant.

## Results

### Comparison of photoreceptor dissociation techniques

**Figure 2:**
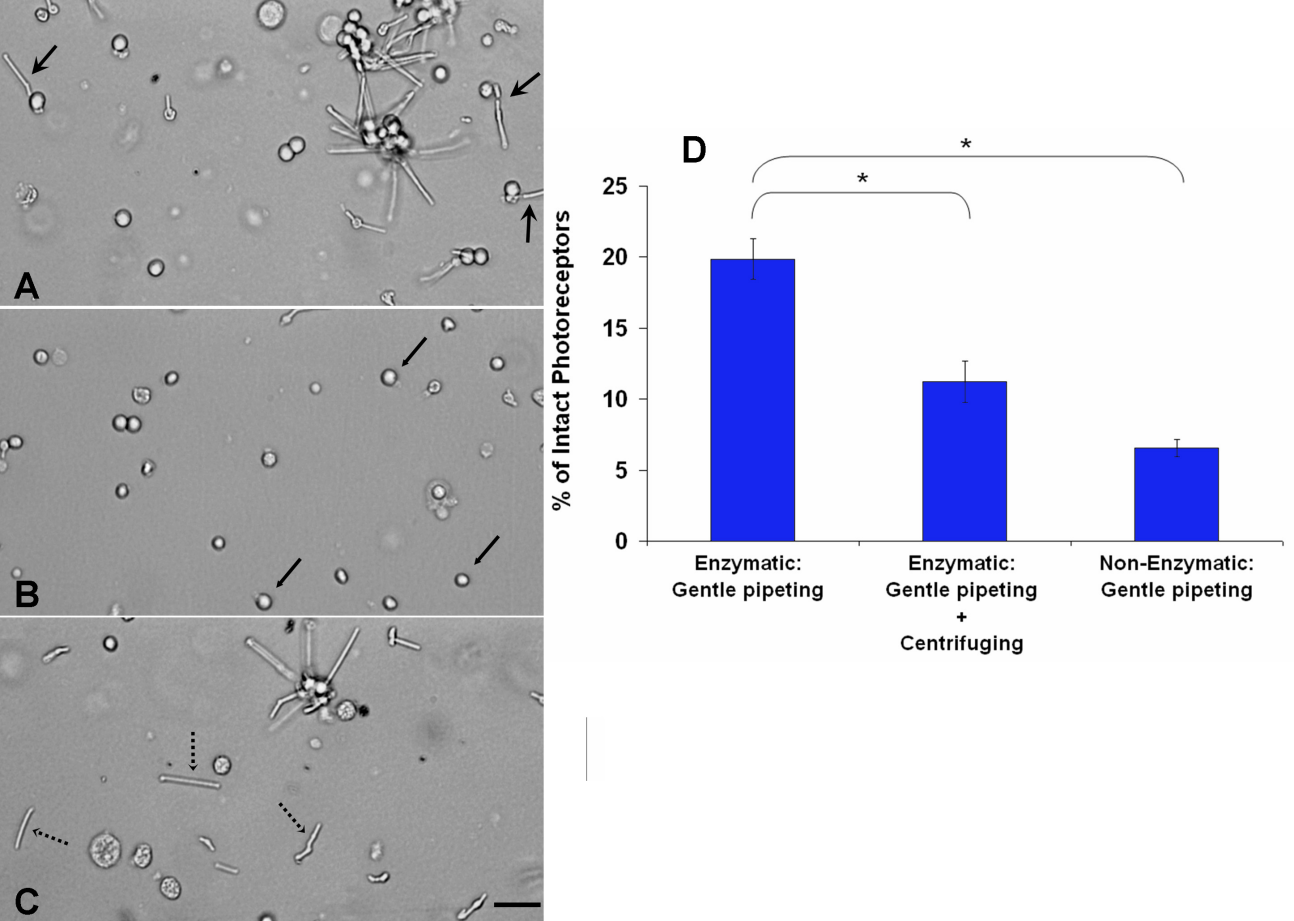
Quantification of intact photoreceptors based on different dissociation techniques. Immediately after seeded in culture, isolated intact photoreceptors were counted under transmitted light. **A:** The enzymatic treatment with gentle pipeting (Protocol A) isolated a large number of intact photoreceptors (arrows with big arch). **B:** The enzymatic treatment with gentle pipeting followed by centrifugation (Protocol B) isolated mostly cell bodies (arrows with small arch). Most of the outer segment structures were lost during the dissociation process. **C:** The non-enzymatic treatment with gentle pipeting (Protocol C) isolated mostly elongated outer segment structures or debris not attached to cell bodies (arrows with dotted line). **D:** Shown are the percentages of intact photoreceptors observed under transmitted light with respect to total of observed nuclei. Protocol A yielded a significantly higher number of intact photoreceptors compared to the other techniques (the asterisk indicates a p<0.001). Error bars represent the standard error of the mean. The scale bar represents 20 μm.

We investigated three standard dissociation protocols that are used for the isolation of adult photoreceptors to determine which protocol yields the highest number of intact cells immediately after dissociation. All of the protocols were performed in room light condition. We defined intact photoreceptors as cells with the outer segment and inner segment still attached to the cell body based on microscopic examination (Figure 1). Protocol A yielded the highest number of intact photoreceptors (Figures 2A,D). At the end of this dissociation, the cell density was mainly composed of intact photoreceptors, photoreceptor cell bodies lacking outer segment, and outer segment debris (Figure 2A). The 19.86±1.43% of the total cell bodies observed were intact photoreceptors. This was calculated to be significantly higher than Protocol B and Protocol C (p<0.001). When the centrifugation step was added to the enzymatic dissociation (Protocol B), the amount of intact photoreceptors decreased to 11.24±1.45% (Figures 2B,D). The cell density obtained with this technique was characterized mostly by rounded cells and outer segment debris. After Protocol C, only 6.56±0.62% of the cell bodies corresponded to intact photoreceptors (Figures 2C,D). The mechanical trituration seemed to be responsible for separating the outer-inner segment from the cell body by breaking the outer fiber. This explains the high number of outer segment debris observed among the cell density (Figure 2C). The results of the non-enzymatic dissociation (Protocol C) confirmed that using papain, or enzymes in general, is necessary for the dissociation of photoreceptor cell bodies from the retina. The enzyme facilitates to break the cell bodies apart from the tight interphotoreceptor matrix and isolate reasonable amounts of intact adult photoreceptors.

**Figure 3:**
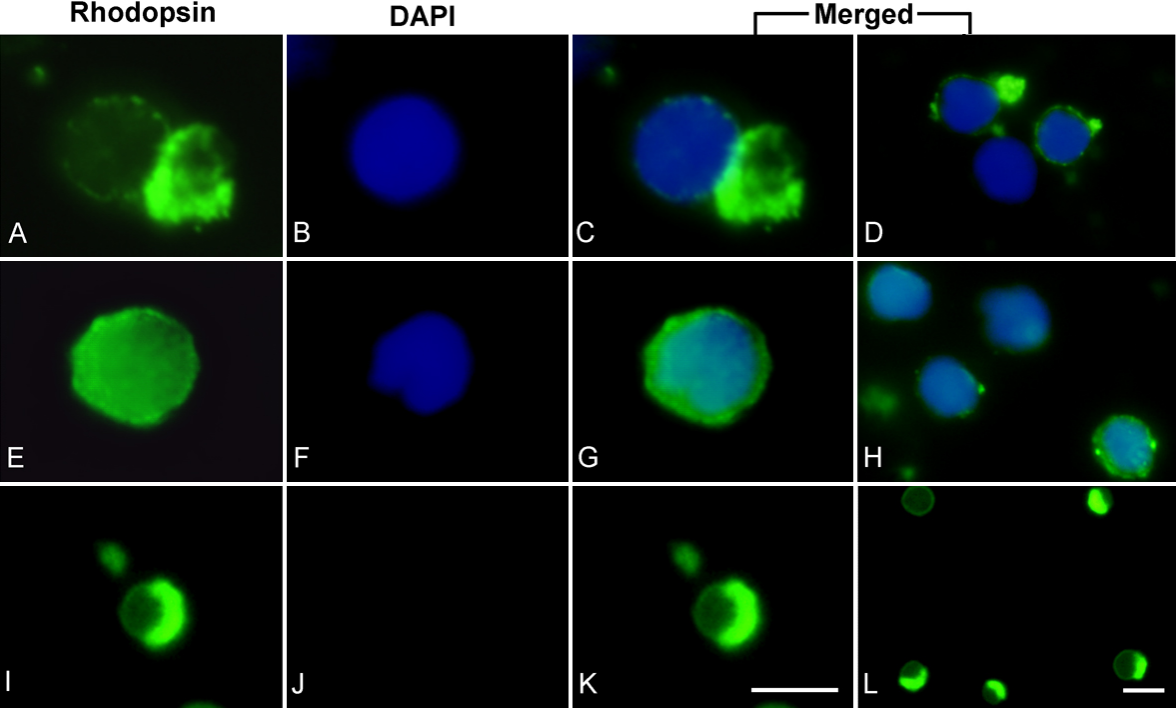
Intact photoreceptors in culture initially dissociated using three different techniques. After 2 days in culture, cells were immunostained with anti-rhodopsin (**A, E, I**; green) for outer segment labeling and with DAPI nuclear staining (**B, F, J**; blue) and observed under epifluorescence illumination. **A-D:** After 2 days in culture, cells dissociated using the enzymatic treatment with gentle pipeting (Protocol A) still showed the outer segment attached to the nucleus/cell body; however, it developed a circular shape. **E-H:** In cells dissociated using the enzymatic treatment with gentle pipeting followed by centrifugation (Protocol B) rhodopsin was mostly expressed within the cytoplasm of the cells. **I-L:** When dissociating the cells with the non-enzymatic treatment with gentle pipeting (Protocol C) rhodopsin expression was observed in outer segment debris. Expression of rhodopsin and DAPI within a same cell was rare. The scale bars represent 5 μm.

These same protocols were used to evaluate the number of intact photoreceptors after 2 days in culture. The experiments were done under regular light room conditions. One difference was that we used the lectin-panning technique, introduced by Balse et al. [25], to promote specific attachment of rod cells by using wheat germ agglutinin coated surfaces. After 2 days, approximately 90% of the intact and elongated photoreceptors obtained using Protocol A had deformed into a rounded shape. However, a deformed outer segment, identified by rhodopsin staining, still remained attached to the nucleus (Figure 3A-D). When Protocol B was used, approximately 90% of the dissociated photoreceptor cell bodies exhibited round shape and lacked outer segment structures. After 2 days of the initial seeding of cells dissociated with Protocol B, the culture was still characterized by round cells (Figure 3E-H). In this case, rhodopsin was mostly expressed within the plasma membrane of the cell body. In cultures obtained using Protocol C, any connection between rhodopsin and nuclei within a same cell was rare. Cultures were characterized by attachment of outer segment debris (Figure 3I-L).

### Effect of light on dissociation technique

**Figure 4:**
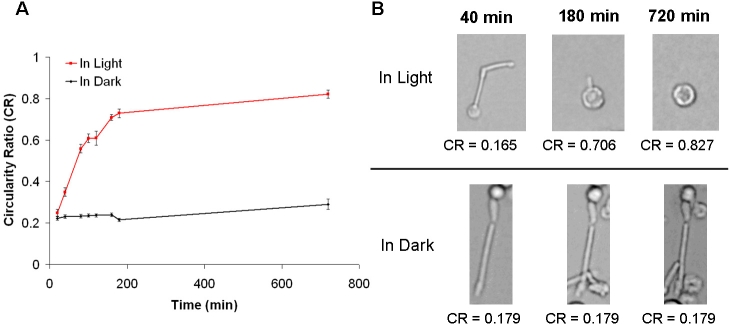
Outer segment elongation as a function of time. Outer segment elongation was measured in cells dissociated using the enzymatic treatment with gentle pipeting (Protocol A) both in light and dark conditions. Time 0 refers to the time of cell seeding. **A:** Light-adapted photoreceptors started to lose their native elongated structure (circularity ratio [CR]>0.2) at 40 min and adopted a circular shape (CR approximately 0.8) at 180 min after seeded in culture. Dark-adapted photoreceptors retained their native elongated shape (CR<0.3) throughout the 720 min of study. Error bars represent the standard error of the mean. **B:** Shown are representative images of light- and dark-adapted photoreceptors.

Our evaluation of photoreceptor dissociation techniques suggested that an enzymatic technique with gentle pipeting (Protocol A) yields a higher number of intact photoreceptors. However, it was observed that the elongated structure of these cells was only temporary, given that by 2 days after seeded in culture, their outer segment were deformed. Therefore, we investigated the time course of the deformation by quantifying the changes in size with time after dissociation both in dark and light adapted conditions. Deformation was analyzed in terms of circularity ratio to separate the circular shape from elongated shape. The values closer to 1 indicated a more circular cell, while values closer to 0 indicated a more elongated cell. We interpreted that elongated cells represent “native” photoreceptor structure. The cells were isolated using the Protocol A in standard room light conditions. Immediately after seeding (time 0), the circularity ratio of the intact cells was approximately 0.25±0.02 and increased to 0.73±0.02 within 180 min after seeding (Figure 4A,B). At 720 min after seeding, the circularity ratio further increased to 0.82±0.02. We observed that in photoreceptors that remained intact after dissociation the outer segments suffered a globular deformation with time. The outer segment tended to curl around the cell body or started fusing into the cell body. After 720 min of study, both intact cells and outer segment debris were rounded.

The experiments were repeated in dark-adapted conditions to investigate if light deprivation prevents deformation of the cell’s outer segment. Under dark-adapted conditions, the amount of intact photoreceptors obtained after the dissociation was approximately 30% higher than the one obtained in light conditions. Also, intact cells remained elongated throughout the duration of the experiment (Figure 4A,B). The average circularity ratio of the cells remained at 0.22±0.01 during the first 180 min and only increased to a maximum value of 0.29±0.03 after 720 min of seeding. After the 720 min of study, most cells were observed to be still intact and elongated.

**Figure 5:**
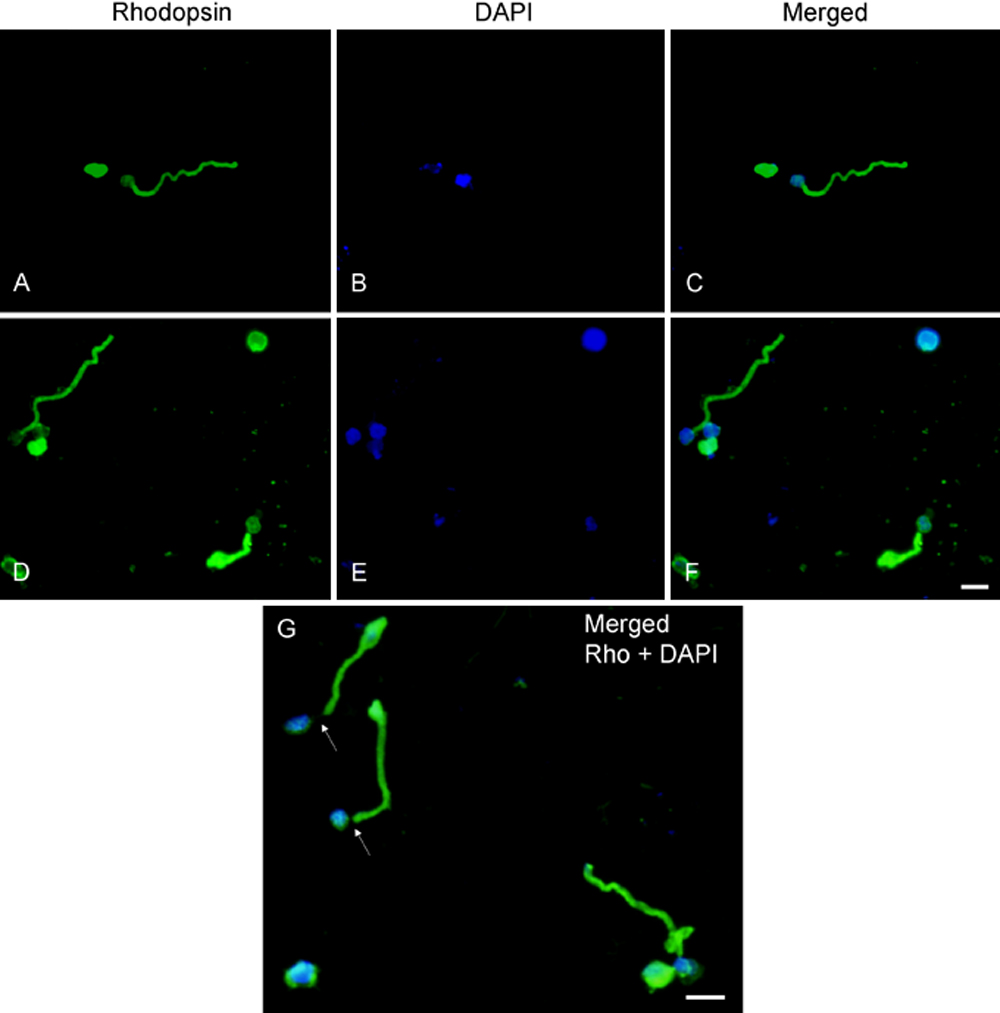
Preservation of intact elongated photoreceptors in culture. Cells were dissociated using the enzymatic treatment with gentle pipeting (Protocol A) in dark-adapted conditions. After 7 days in culture, cells were visualized under epi-fluorescence illumination after immunostaining with anti-rhodopsin (**A, D**; green) and DAPI nuclear staining (**B, E**; blue). Merged images of rhodopsin (Rho) and DAPI (**C, F**, and **G**) showed the elongated outer segment still attached to the nucleus. Some rounded cells were still observed; but cells were, in majority, elongated. **G:** Arrows indicate the outer fiber connecting the outer-inner segment to the nucleus. Scale bars represent 5 µm.

To investigate the preservation of the elongated structure of photoreceptors, we examined cells by immunohistochemistry after 7 days in culture. Cells were initially isolated using Protocol A in dark adapted conditions and covered from light even after seeded. Medium changes were done in darkness. We observed that most photoreceptors remained intact and elongated after 7 days. Immunolabeling with rhodopsin showed that the outer segment was still attached to the cell body and was well preserved (Figure 5).

### Viability of photoreceptors with time

**Figure 6:**
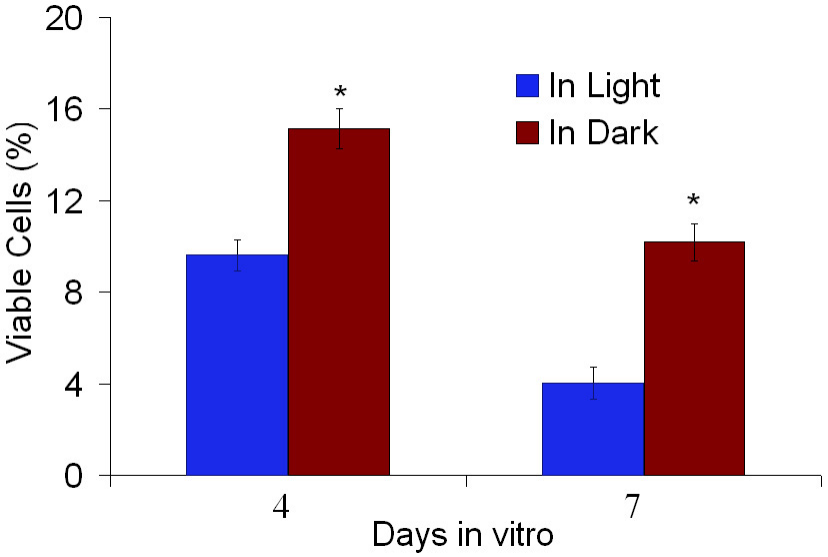
Comparison of cell viability after 4 and 7 days in vitro. The percent of the initial number of cells seeded that remain viable and attached was measured using the CellQuanti-Blue™ assay both in light (blue) and dark (red) culture conditions. The percent of cells that remain viable was significantly higher in experiments performed in dark-adapted conditions (the asterisk indicates a p<0.001). Error bars represent the standard error of the mean.

We quantified the number of attached cells that remained viable after 4 and 7 days in vitro both in dark and light conditions using a cell-viability assay that counts the amount of viable cells based on their metabolic function during the reduction of resazurin (CellQuanti-Blue™ cell viability assay). The number of cells at each time point was compared to the number of cells initially seeded to obtain the percent of viable cells. Both after 4 and 7 days in vitro, the number of viable-attached cells was significantly higher (p<0.001) in cultures performed in dark-adapted conditions (Figure 6). After 4 days, 15.13±0.89% of the initial number of cells seeded remained viable and attached compared to 9.61±0.68%. After 7 days, the numbers decreased to 10.19±0.81% of viable cells in dark conditions and 4.02±0.70% in light conditions. The decrease of viable cells between day 0 and day 4 are mostly due to low attachment of initially seeded cells, which are washed out during medium replacement.

## Discussion

Cell culture of fully developed and functional adult photoreceptor cells can serve as an in vitro model for the study of retinal diseases and the search of prospective therapies. However, obtaining cells that resemble identical characteristics as in vivo cells is still a major challenge. In long-term cultures, photoreceptors have been observed to undergo morphological changes by suffering of outer segment loss and becoming rounded as shown in this study as well as previous studies [10,23,24]. In this study, we have evaluated the current available techniques for adult photoreceptor dissociation to determine which one yields the highest number of intact photoreceptors. We also examined the timeframe of the deformation of intact cells after seeded and the possible contribution of light to this deformation.

Our results showed that the optimal technique to obtain a high number of intact elongated photoreceptors was an enzymatic dissociation followed by a gentle mechanical trituration (Protocol A). Not using enzymes, as in Protocol C, prevents from breaking the cell bodies apart from the tight interphotoreceptor matrix and isolating individual intact photoreceptors. A gentle enzymatic technique consists in exposing the retinal tissues to low doses of papain (0.06–0.1 mg/ml) for short 20 min incubation [4,16]. In these experiments, we refrained from activating papain with L-cysteine as it has been shown to be cytotoxic [31]. We also minimized handling of the tissues by using only wide bore pipette tips and avoiding any agitation of tubes containing the tissue. Our data also suggested that centrifugation after the dissociation to obtain a cell pellet should be avoided as the mechanical stress seemed to contribute to cell deformation. Based on the data, it should be noted that the centrifugation step should be minimized or eliminated from the protocol.

Immunohistochemistry results showed that 2 days after seeding, cells had deformed into a circular shape, even though cells were intact and elongated when initially seeded. The outer segment was still observed attached to the cell body/nucleus but deformed from elongated to round in shape (Figure 3A-D) and this observation is consistent with Townes-Anderson et al. [22]. Cells that had lost their outer segment during the dissociation process expressed rhodopsin, but within their plasma membrane (Figure 3E-H), as previously observed [4,10,23,35]. The time course of the deformation demonstrated that it took approximately 3 h after seeding for all intact cells and outer segment debris to become rounded (Figure 4A,B). Based on our data, a gentle handling and dissociation are key factors to maintain high numbers of cells. However, the cell deformation cannot be eliminated based on the handling and dissociation factors alone.

To preserve as much elongated photoreceptors in culture, we investigated whether the light exposure during dissociation and culture process play a role in the deformation of outer segments. There are no direct studies to date that have investigated the effect of light on outer segment deformation. However, it has been shown that deformations have been observed in outer segments as a result of changes in osmotic pressure [36-38]. Under hypertonic saline solutions, isolated outer segments shrank in size and shape in response to light. It was hypothesized that the change was due to the closing of cGMP-gated channels in response to light [36,37]. Similarly, hypotonic solutions caused outer segments to deform and become rounded after cultivation for 10 min under either light or dark conditions [38]. Hypotonic solutions are known to hyperpolarize cell membranes [39-41], a phenomenon that also occurs in photoreceptors in response to light. In a comparison of our study with microscopic images from Cohen [38], we noted a similar cell deformation; this may suggest that light exposure may play a role in altering the cell shape in culture. Therefore, it is possible that deformations caused by light conditions as well as hypotonic solutions are related, although more research should be performed to correlate these two phenomena.

Our preliminary results showed that photoreceptors isolated in dark conditions better maintained their elongated shape even 12 h after seeding, and remained in an elongated shape for 7 days. In contrast, photoreceptors isolated under room light conditions changed the shape within 3 h, with its deformation starting about 45 min after seeding. Our data suggested that there may be a relationship between the photoreceptor’s ability to maintain structure and light exposure during the dissociation process. Although our results seemed to favor our hypothesis, further research should be conducted to explore the possible mechanism of light involvement and duration in which elongated cells can be maintained. A viability assay after 4 and 7 days in culture indicated that performing experiments in dark promoted higher number of cells to remain viable with time. Based on our data, well preserved elongated cells can be viable for a longer period of time if light exposure is minimized or eliminated during the dissociation and culture process.

The current study demonstrated two important characteristics of the isolation and culture of photoreceptor cells. Our results showed the possibility of using adult photoreceptors maintained in vitro for the experiments mentioned in the Introduction and in transplantation techniques.

### Future plans

The results of this study open many questions regarding the mechanisms that minimize cell deformation in dark conditions. The use of channel blockers to test for possible mechanisms involved is a valuable avenue being considered for immediate future studies. If cell deformation is observed in the dark by blocking cGMP channels, we may be able to suggest that cGMP channels are one of the main factors involved. If no deformation occurs by blocking cGMP channels, we cannot rule out completely the involvement of cGMP channels without testing other channels (e.g., K^+^ channels or hyperpolarizing-activated channels). Therefore, an in-depth pharmacological studies considering involvement of cGMP channels and additional outer and inner segment channels is essential and will be highly informative.
